# Brain activity and autonomic regulation in untreated migraine patients

**DOI:** 10.1055/s-0045-1808085

**Published:** 2025-06-17

**Authors:** Isabel Oliveira Monteiro, Juliana Novakovic, Miguel K. Rodrigues, Joel S. Cunha Neto, Maíra O. V. Rela, Mayron F. Oliveira

**Affiliations:** 1 Universidade de Fortaleza, Centro de Ciências da Saúde, Curso de Fisioterapia, Fortaleza CE, Brazil.; 2 Lyon College, Exercise Science Department, Exercise Physiology and Integrated Cardiopulmonary Research Group, Batesville AR, United States.; 3 VO2 Care, Unidade de Fisioterapia, São Paulo SP, Brazil.; 4 Universidade de Fortaleza, Centro de Ciências Tecnológicas, Fortaleza CE, Brazil.

**Keywords:** Migraine Disorders, Brain Mapping, Heart Rate, Autonomic Nervous System

## Abstract

**Background:**

Migraine causes intense pain, significant disability, deficits in attention and memory, slowed information processing, and cognitive disorders. However, it is unclear how migraine, cardiovascular, and cerebral issues impact daily life or relate to future adverse events.

**Objective:**

To evaluate the brain activity and autonomic regulation in untreated migraine patients.

**Methods:**

In the present case-control study, we compared untreated migraine patients with healthy controls. The participants underwent cognitive testing (Stroop Color Test, Trail Making Test, Addenbrooke's Cognitive Examination, and Reaction Time Test), brain activity measurement (MindWave Mobile), and autonomic regulation assessment via heart rate variability (Polar V800).

**Results:**

No differences were found between the groups in terms of cognitive test scores. However, the healthy controls consistently showed increased variation in brain activity during the cognitive tests, while migraine patients exhibited decreased activity across all tests ( *p* < 0.05). During the Stroop Color Test, the controls showed a positive change in brain activity (Δ = 5.12 ± 3.64) while the migraine patients showed a negative change (Δ = −5.41 ± 2.21). In addition, the control group demonstrated a normal autonomic response, with increased sympathetic activity (low-frequency [LF] band: 70.2–84.4 Hz) and decreased parasympathetic activity (high-frequency [HF] band: 29.6–15.6 Hz) during cognitive tasks ( *p* < 0.05). In contrast, the migraine group showed imbalanced autonomic regulation, characterized by minimal changes in both sympathetic (LF band: 74.0–74.8 Hz) and parasympathetic activity (HF band: 25.9–25.1 Hz) ( *p* > 0.05).

**Conclusion:**

Despite the similar cognitive test scores, migraine patients exhibited reduced variation in brain activity during cognitive tests and an imbalanced autonomic regulation, characterized by decreased sympathetic activity and increased parasympathetic activity.

## INTRODUCTION

Patients with migraines experience cognitive and mental effects that negatively affect their productivity and performance in daily activities. This relationship is reported by the patients themselves in the form of attention and memory deficits, slowed speed in information processing, and mental disorders that contribute to negative changes in the productivity and performance in their activities. 1 2 3

The pathophysiology of migraine involves a complex interplay between neurological and vascular mechanisms, triggering a cascade of events that affect multiple brain regions. Recent studies 1 4 5 have demonstrated that migraine is not merely a pain disorder, but a complex neurological condition with significant implications for autonomic nervous system (ANS) regulation. This widespread neuronal dysfunction is not limited to pain-associated areas, but also impacts regions responsible for higher cognitive functions. 6 Consequently, migraine patients frequently report a range of cognitive symptoms, including difficulties in concentration, memory lapses, and slowed information processing.

Thus, patients with migraine might report symptoms of cognitive disorders, so it is also necessary to investigate the possible existence of mental alterations related to the clinical condition. 3 7 In addition to the possibility of being closely related to cognitive dysfunctions, many studies also hypothesize the influence of migraine on systemic changes, including the ANS, which, when affected by stress, can lead to increased headaches due to sympathetic dysregulation. Additionally, migraines have been proposed 8 9 to impact the vascular system through homeostatic changes.

It has been suggested 9 that migraine acts on the vascular system through changes in its homeostasis, predisposing to the occurrence of unbalanced equilibrium between vasoconstriction and/or vasodilation. Emerging research 10 11 12 13 14 has revealed critical insights into the autonomic dysregulation associated with migraine; these studies underscore the importance of comprehensive evaluations that extend beyond traditional pain-focused approaches, revealing intricate connections involving neural pathways, cardiovascular responses, and cognitive processing in migraine patients. Moreover, the close relationship between the neural pathways involved in migraine and autonomic control centers suggests a potential mechanism for the observed alterations in cardiovascular regulation in these patients. By integrating these perspectives, a more nuanced understanding of the multisystemic nature of migraine emphasizes the need for holistic research methodologies that capture the complex interplay among neurological, autonomic, and cognitive domains.

While many studies have reported cognitive impairment in migraine subjects, the literature presents a more complex picture. Jelic et al. 15 (2000) found that migraine patients performed worse on tests of sustained attention and verbal memory. However, larger community-based studies have yielded different results. Gaist et al. 16 (2005) conducted a large population-based study and found no significant differences in cognitive function between middle-aged migraine patients and controls. Similarly, in a longitudinal study, Wen et al. 17 (2016) reported no increased risk of cognitive decline in elderly migraine sufferers compared with non-migraine individuals. Interestingly, Gil-Gouveia and Martins 18 (2019) noted that some studies even showed better cognitive performance in migraine patients during the interictal period. These conflicting findings suggest that the relationship between migraine and cognitive function may be more nuanced than previously thought, and it could be influenced by factors such as study design, sample selection, and disease severity.

However, it is unclear how and if migraine, cardiovascular, and cerebral disorders could affect daily life. To date, few studies have evaluated the ANS and brain activity in migraine subjects, but no paper has evaluated untreated migraine subjects. Based on this, we hypothesized that young adults with untreated migraine would exhibit cognitive impairment, such as decreased brain activity and memory, with an impaired ANS regulation compared with a healthy group. The present study aimed to evaluate cognitive function, brain activity, and autonomic regulation in untreated migraine patients compared with healthy controls.

## METHODS

A cross-sectional case-control study was performed at Universidade de Fortaleza (UNIFOR), in the state of Ceará, Brazil. The study included 80 individuals: 40 in the control group and 40 in the migraine group.

The migraine volunteers and healthy controls were recruited among students and employees of UNIFOR, including its Integrated Medical Care Center (Núcleo de Atenção Médica Integrada, NAMI, in Portuguese). Messages were posted on social media and posters were put up around the campus, explaining the study objectives and inviting eligible volunteers to participate. The controls were additionally screened by the occupational health physician to confirm their health status. This approach ensured similar educational backgrounds across the groups, as all participants were associated with the university environment.

We enrolled male and female individuals aged ≥ 18 years with untreated migraines, who had received a previous clinical diagnosis of migraine from a medical doctor according to The International Classification of Headache Disorders. 19 Migraine volunteers were paired with healthy controls of a similar age without a history of migraines. Participants in any of the groups were excluded if they had any cardiovascular comorbidities (type-I and -II diabetes, hypertension, previous cardiac surgery, heart failure, vascular insufficiency, or arterial calcification), cognitive problems or dysfunction associated with cognition, a history of stroke or transient ischemic attack, or any visual problems that compromised test performance.

The participants were submitted to assessments regarding data on personal background, cognitive function, brain activity, and heart rate variability (HRV). The cognitive tests were administered in a randomized order determined by a draw. All participants were pain-free at the time of data collection and reported no migraine attacks for at least 5 days before the assessment. This criterion was implemented to exclude the prodromal period, which can occur up to 48 hours before pain onset and potentially confound cognitive assessments. The current study was approved by the UNIFOR Ethics in Research Committee (under nr. 3.741.233), and written informed consent was obtained from all participants.

Brain activity was recorded by MindWave Mobile during all tests, with an assessment of the HRV (using the Polar V800 heart rate monitor). Baseline HRV and brain activity data were recorded after a 5-minute resting period before conducting the tests and also during all tests, which were performed in the morning to maintain stable hormonal and circadian cycle regulation. Data were also analyzed in variations (final data – baseline data [Δ]).

### Cognitive tests

The following validated tests were used to assess cognitive function: Stroop Color Test 20 (SCT, a psychological tool designed to measure cognitive abilities related to attention, processing speed, and cognitive flexibility), Addenbrooke's Cognitive Examination (ACE, a neuropsychological test designed to measure various cognitive domains, including attention, memory, language, and visuospatial skills), 21 Reaction Time Test 22 (RTT, psychomotor assessment designed to measure the speed at which an individual responds to a specific stimulus), Trail Making Test A (TMTA, which measures attention, visual scanning, and psychomotor speed), and Trail Making Test B (TMTB, which measures cognitive functions, including cognitive flexibility, task switching, and visual-motor coordination). 23

### Brain activity

The NeuroSky MindWave Mobile headset (henceforth, MindWave) was the device used to measure brain activity (beta [β] wave) using a single electrode placed on the forehead, which was validated elsewhere. 24

The device uses a monopolar montage, meaning it has one active site and a small electrode clipped to the left earlobe as a reference. This device has an electrode functioning as a sensor to detecting brainwave signals, interpreting them through software (Brainwave Visualizer – brain activity data), and it uses only one passive dry electrode on the forehead to measure brain functions. 24 Some potential applications include monitoring brain activity, assessing cognitive function, detecting neurological disorders, and developing brain-computer interfaces.

### Heart rate variability

The autonomic function was recorded using the HRV (Polar V800) to analyze the R-R (HRV is analyzed using two R's waves in the ECG) intervals corresponding to the cardiac cycle at baseline and during the entire evaluation. The Polar device estimates presented a good intraclass correlation and relatively narrow limits of agreement at lower electrocardiogram-derived values, making it useful as an HRV monitor and for physiological studies. 25

The analysis of the HRV spectrum for each test was performed using the Kubios software. Briefly, the analysis of the HRV based on frequency domains involves the measurement of the power spectral density of the heart rate signal. The high-frequency (HF) band (0.15–0.4 Hz) reflects parasympathetic activity (vagal influence), while the low-frequency (LF) band (0.04–0.15 Hz) reflects/considers the performance of the sympathetic system. The ratio between the LF and HF (LFHFr) power is often used as an index of sympathetic-vagal balance. 25

### Statistical analysis

We used the IBM SPSS Statistics for Windows software, version 20.0, for the statistical analysis, which included descriptive statistics such as mean, standard deviation, relative frequency, and absolute frequency values. No statistical power calculation was conducted before the study, and the sample size was based on our previous experience with this design. Normality was assessed using the Shapiro-Wilk test, and comparisons between groups were made using the Chi-squared test for categorical data, the Student's *t* -test for independent samples, and the paired *t* -test for intragroup samples. Statistical significance was defined as *p* < 0.05. Additionally, Cohen's d effect sizes were calculated for key comparisons, including brain activity during the SCT and HRV (LF and HF) changes during cognitive tasks, to better contextualize the magnitude of the differences observed between groups.

## RESULTS

A total of 80 individuals participated in the study: 40 in the control group and 40 in the migraine group. All participants were female. Additionally, the control and migraine groups were similar in terms of the rates of previous comorbidities (such as alcohol consumption [20%], smoking [10%], and hypertension [20% versus 30% respectively]; Table 1 ). No migraine medication intake was reported in either group.

**Table 1:** Baseline characteristics and cognitive tests of untreated migraine volunteers and healthy controls

		Control ( *n* = 40)	Migraine ( *n* = 40)	*p* -value
***Anthropometry/Demography***	Age (years)	35 ± 4	33 ± 5	0.991
	Weight (kg)	67 ± 3	59 ± 5*	0.040
	Height (m)	1.60 ± 0.02	1.61 ± 0.01	0.768
	BMI (kg/m ^2^ )	24.3 ± 2.3	22.8 ± 1.6	0.057
	Physical activity: n (%)	36 (90%)	28 (70%)	0.518
***Comorbidities***	Smoking: n (%)	4 (10%)	4 (10%)	0.988
***Cognitive tests***	SCT time (s)	118.8 ± 3.2	113.3 ± 3.1	0.250
	SCT score (a.u.)	103.0 ± 5.0	101.0 ± 5.0	0.435
	TMTA time (s)	34.7 ± 3.3	53.1 ± 6.6	0.055
	TMTB time (s)	74.9 ± 15.0	74.3 ± 13.2	0.870
	RTT time (s)	37.3 ± 1.5	35.8 ± 2.0	0.641
	ACE time (s)	633.4 ± 19.6	614.9 ± 76.6	0.690
	ACE score (a.u.)	92.2 ± 2	91.7 ± 4.0	0.779

Abbreviations: ACE, Addenbrooke Cognitive Examination; a.u., arbitrary units; BMI, Body Mass Index; RTT, Reaction Time Test; SCT, Stroop Color Test; TMTA, Trail Making Test A; TMTB, Trail Making Test B.

Notes: Values are expressed as: a mean ± standard deviation; and n and percentage. * *p* < 0.05 between groups.

When the data for response time and scores on the cognitive tests were evaluated, no differences were found between the groups ( Table 1 ). However, the mean time taken to perform the TMTA test was shorter in the control group (34.7 ± 3.3 seconds) compared with migraine group (53.1 ± 6.6 seconds). In addition, the brain activity variation (final data – baseline data [Δ]) values at the end of the cognitive tests were significantly different for all tests performed. The brain activity (β waves) changes were physiologically enhanced in the controls; Nevertheless, a significant decrease in brain activity (β waves) was observed in the migraine group during the tests ( Table 2 ).

**Table 2:** Variations in brain activity before and after the application of cognitive tests

Δ Brain activity (beta waves)	Control ( *n* = 40)	Migraine ( *n* = 40)	*p* -value
Stroop Color Test, points (a.u.)	5.2 ± 3.4	-5.1 ± 1.9*	0.02
Trail-Making Test A, points (a.u.)	9.2 ± 4.6	3.4 ± 1.3*	0.01
Trail-Making Test B, points (a.u.)	1.4 ± 0.2	-11.1 ± 1.3*	0.01
Reaction Time Test, points (a.u.)	16.3 ± 6.1	-12.6 ± 4.9*	0.01
Addenbrooke's Cognitive Examination, points (a.u.)	7.3 ± 3.5	-13.7 ± 5.8*	0.01

Abbreviation: a.u., arbitrary units.

Notes: Values are expressed as a mean ± standard deviation. Δ is the difference between the final data and baseline data. * *p* < 0.05 between groups.

Notable differences in HRV responses were observed between both groups, which was an interesting finding. The control group showed a physiological response regarding parasympathetic withdrawal associated with an increase in sympathetic activity compared with the migraine group ( Table 3 ). On the other hand, the migraine group presented a reduced improvement in sympathetic activity while the parasympathetic remained the same as baseline.

**Table 3:** Values of heart rate variability before and after the application of cognitive tests

		Baseline		Final	
		Control ( *n* = 40)	Migraine ( *n* = 40)	Control ( *n* = 40)	Migraine ( *n* = 40)
**Stroop Color Test**	HF, a.u.	29.6 (15.1–44.2)	25.9 (15.5–36.3)	26.9 (10.4–43.3)	23.0 (14.6–31.4)
	LF, a.u.	70.2 (55.4–84.9)	74.0 (63.5–84.5)	72.7 (56.0–89.4)	77.0 (68.5–85.4)
	LFHFr, a.u.	4.0 (0.4–7.5)	4.0 (1.8–6.3)	4.3 (0.6–8.0)	4.9 (1.9–7.0)
**Trail-Making Test A**	HF, a.u.	29.6 (15.1–44.2)	25.9 (15.5–36.3)	26.0 (8.9–43.1)	27.7 (18.0–37.3)
	LF, a.u.	70.2 (55.4–84.9)	74.0 (63.5–84.5)	73.9(56.5–91.2)	72.3 (62.7–82.0)
	LFHFr, a.u.	4.0 (0.4–7.5)	4.0 (1.8–6.3)	4.8 (1.4–8.2)	3.2 (2.0–4.4)*
**Trail-Making Test B**	HF, a.u.	29.6 (15.1–44.2)	25.9 (15.5–36.3)	30.7 (8.4–53.0)	23.2 (9.8–36.7)*
	LF, a.u.	70.2 (55.4–84.9)	74.0 (63.5–84.5)	69.1(46.6–91.7)	76.8 (63.3–90.2)*
	LFHFr, a.u.	4.0 (0.4–7.5)	4.0 (1.8–6.3)	4.8 (0.1–9.6)	6.3 (1.8–10.7)*
**Reaction Time Test**	HF, a.u.	29.6 (15.1–44.2)	25.9 (15.5–36.3)	15.6 (6.9–24.3)†	25.1 (11.2–40.0)*
	LF, a.u.	70.2 (55.4–84.9)	74.0 (63.5–84.5)	84.4 (75.7–93.1)*†	74.8 (60.9–88.6)*
	LFHFr, a.u.	4.0 (0.4–7.5)	4.0 (1.8–6.3)	8.7(1.6–15.8)*†	4.9 (2.4–7.4)*
**Addenbrooke Cognitive Examination**	HF, a.u.	29.6 (15.1–44.2)	25.9 (15.5–36.3)	22.0 (14.6–29.4)†	22.6 (17.1–28.0)
	LF, a.u.	70.2 (55.4–84.9)	74.0 (63.5–84.5)	77.6 (69.8–85.3)†	77.3 (71.8–82.9)
	LFHFr, a.u.	4.0 (0.4–7.5)	4.0 (1.8–6.3)	4.0 (2.5–5.4)	4.2 (2.1–6.2)

Abbreviations: a.u., arbitrary units; HF, high frequency; LF, low frequency; LFHFr, low frequency high to frequency ratio.

Notes: Values are expressed as median (interquartilerange). * *p* < 0.05: control group versus migraine group. ^†^ *p* < 0.05 baseline data versus final data.

We calculated effect sizes to better contextualize our findings. For brain activity during the SCT, the control group showed a positive change (Δ = 5.12 ± 3.64) while the migraine group exhibited a negative change (Δ = −5.41 ± 2.21), with a large effect size (Cohen's d = 3.48). Regarding HRV, the control group demonstrated significant changes in the LF (70.2–84.4 Hz) and HF (29.6–15.6 Hz) components during cognitive tasks. In contrast, the migraine group showed minimal changes in LF (74.0–74.8 Hz) and HF (25.9–25.1 Hz). The effect size for LF changes between groups was large (Cohen's d = 1.12).

## DISCUSSION

The present study demonstrated a physiological response/increase in brain activity and sympathetic activity in the control group when faced with stress tests and impaired-to-reduced brain activity and an unbalanced ANS response (sympathetic and parasympathetic activity) in the migraine group.

In the current study, no significant differences were found between migraine patients and controls in terms of cognitive test scores. However, migraine patients exhibited a distinct pattern of brain activity reduction during cognitive tasks, while healthy controls showed increased brain activation. Additionally, autonomic regulation was impaired in the migraine group, with reduced sympathetic response and unchanged parasympathetic activity. Our findings of cognitive differences between migraine patients and controls align with those of certain previous studies but contrast with those of others. This discrepancy might be explained by the heterogeneity among migraine populations and study designs. As noted by Gil-Gouveia and Martins 18 (2019), studies conducted in specialized headache clinics often report more pronounced cognitive impairment compared with community-based studies. This suggests that clinic-based samples may represent more severe forms of migraine, potentially overestimating the cognitive impact of migraine in the general population. The results of the present study, showing subtle cognitive differences and significant changes in brain activity and autonomic regulation, highlight the importance of considering multiple physiological parameters when assessing the impact of migraine. Future research should aim at reconciling these conflicting findings by conducting large-scale, community-based studies that incorporate both cognitive assessments and physiological measurements.

One of the possible ways to trigger migraine would be the excitation of the hypothalamus through stressful external stimuli, such as cognitive tests and the RTT, which can initiate a hormonal release response acting in the ANS pathways. 5 This may lead to the release of parasympathetic neurotransmitters with vasodilating action, such as acetylcholine and nitric oxide, in the sphenopalatine ganglion, originating an inflammatory cascade with the potential of activating meningeal nociceptors. 4 26 27 Therefore, the interaction between the ANS and the sensory system of the trigeminal nerve generated by the hypothalamic activation would supposedly facilitate the advancement of the migraine process by altering the balance of the sympathetic and parasympathetic performances. 26 28 Indeed, it is known that the body's primary physiological response to stressful stimuli should be the intensification of the action of the sympathetic nervous system, which could be observed in the control group for most of the tests applied in the current study. However, the absence of response in the migraine group suggests that an additional mechanism could be involved in the ANS in patients with migraine. 9 29

The lack of response in the migraine group suggests an additional mechanism involved in the ANS of these patients. While the control group showed an expected intensification of sympathetic nervous system action in response to stressful stimuli, the migraine group did not exhibit this typical response. This indicates that there may be an additional or altered mechanism in the functioning of the ANS in migraine patients, which could contribute to the pathophysiology of the condition. 8

The alterations in brain activity observed in migraine patients have a complex and multifaceted relationship with neural processing, involving structural, functional, and regulatory aspects of the brain. Previous neuroimaging studies have demonstrated that abnormal functional connectivity in brain regions involved in cognitive processing is associated with impaired performance on attention and executive function tasks in migraine patients. However, in the present study, despite the significant reduction in brain activity variation in the migraine group, no differences were observed in cognitive test performance between groups. This suggests that changes in brain activity and autonomic regulation may occur even in the absence of measurable cognitive deficits. Furthermore, structural changes in the brains of migraine patients have been identified and may be related to cognitive function, including reduced gray matter volume in areas associated with cognition. Gil-Gouveia et al. 30 (2015) proposed that cognitive symptoms during migraine attacks may be both a consequence of and a trigger for pain, highlighting the complex interplay between brain activity and cognition. The dysregulation in the autonomic nervous system observed in the current study may also play a role in this process. Previous papers 11 12 31 32 have reported that reduced HRV in migraine patients was associated with poorer performance on tests of attention and processing speed. This relationship between autonomic function and cognition is corroborated by our findings of imbalanced autonomic regulation during cognitive tasks in migraine patients. While the present study did not detect differences in cognitive test scores, the observed alterations in brain activity and autonomic regulation may still have implications for long-term cognitive health. The complex interplay among structural, functional, and regulatory aspects of the brain in migraine patients underscores the need for a comprehensive approach to understand and monitor potential cognitive alterations in this population. 12 30 33 34

Previous studies 35 have demonstrated an association between migraines and a higher risk of dementia. The current study opens new possibilities for long-term research follow-up in the assessment of cognition/brain activity in individuals with migraine and its long-term effects, such as possible Alzheimer's disease and/or dementia, 35 36 as well as other pathologies related to the cerebral vascular system. In this perspective, a previous study 31 stated that possible changes in cerebral perfusion related to its pathogenesis would be linked to cognitive abnormalities observed in individuals with migraine. On the other hand, Ogoh 37 showed that changes in cerebral vascularization did not affect cognition and prioritized the hypothesis of the action of the brain's metabolism as a physiological factor determining cognitive performance in migraine patients. Therefore, it is clear that, even though it does not correspond to its typical symptoms, the decline in cognitive function is not infrequent in the context of migraine; thus, more studies are needed to clarify the means for its occurrence and its possible impacts. 8 18 30 While the current study provides valuable insights into brain activity, cognitive alterations, and autonomic regulation in untreated migraine patients, it is important to note that the potential mechanisms underlying these alterations have not been fully elucidated in the scope of our current protocol.

As a cross-sectional, case-control study, the design of the present study primarily focuses on assessing the existing differences between untreated migraine patients and healthy controls. Regarding the progression of temporary results to chronic conditions, we acknowledge that longitudinal cohort studies would be essential to explore such dynamics. The study highlights the potential long-term effects of migraine on cognition, including possible links to dementia and Alzheimer's disease. The results suggest that alterations in cognitive function, although not typical symptoms of migraine, are not infrequent in this context. The study opens new possibilities for long-term follow-up research in the assessment of cognition and brain activity in individuals with migraine. Additionally, it raises questions about how changes in cerebral perfusion or brain metabolism might be related to the cognitive abnormalities observed in individuals with migraine. 8 While our findings highlight trends toward changes in cognitive function in untreated migraine patients, we emphasize the need for further comprehensive investigations, such as cohort studies, to better understand the potential long-term implications of recurrent headache attacks on cognitive outcomes. The association between migraines and cognitive decline is a complex and multifaceted phenomenon that warrants in-depth exploration in future research endeavors.

Our results suggest that the lower scores in brain activity associated with lower HRV in the migraine group could be an earlier sign of a worst-case scenario. However, it has been previously stated that migraine patients, if medicated, have been seen to experience a delay in the dementia and or mental/cognitive symptoms. The lack of significant HRV changes in migraine patients during the SCT and ACE, as opposed to the other tests, may be attributed to the specific cognitive domains these tests assess and their structure. The SCT measures selective attention and cognitive flexibility, while the ACE is a comprehensive cognitive screening tool. Critchley et al. 38 (2003) demonstrated that different cognitive tasks can have varying effects on autonomic responses, including HRV. Tasks requiring sustained attention and working memory, which are prominent in these tests, may elicit different autonomic responses compared with other cognitive tasks. Moreover, the continuous nature of these tests, without distinct breaks between subtasks, might result in a more stable autonomic state throughout the assessment. This contrasts with tests with clear transitions that could trigger more noticeable autonomic shifts. Vuralli et al. 8 (2018) noted that migraine patients often show altered patterns of brain activation during cognitive tasks, which could influence autonomic responses to these specific tests. Further research comparing autonomic responses to various cognitive tests in migraine patients could help elucidate these differences. While the current study demonstrates robust internal validity due to careful participant selection and matching in a case-control design, we acknowledge the need for caution in generalizing the results. The study focused on a specific subset of untreated migraine patients within a “young” age group, and the findings may not necessarily extend to all migraine patients or diverse age ranges. Treated migraine patients often show improved autonomic regulation and reduced migraine severity compared with untreated patients. Prophylactic treatments can enhance HRV and stabilize autonomic function. 39 Untreated migraine patients exhibit more pronounced autonomic dysregulation, evidenced by lower HRV and imbalanced sympathetic and parasympathetic activity. This dysregulation can exacerbate migraine symptoms and contribute to chronicity. 40 Our findings of impaired autonomic responses in untreated migraine patients align with those of previous research 41 indicating heightened sympathetic response and reduced parasympathetic activity in these individuals. Future research should compare autonomic regulation in treated versus untreated migraine patients to better understand treatment effects on autonomic function and optimize strategies to improve autonomic balance.

Our findings provide new insights into the autonomic dysregulation associated with migraine. The present study shows that migraine patients exhibited an imbalanced autonomic regulation, characterized by minimal changes in both sympathetic and parasympathetic activity during cognitive tasks, in contrast to the normal autonomic response observed in healthy controls. These findings underscore the importance of comprehensive evaluations that extend beyond traditional pain-focused approaches, revealing intricate connections involving neural pathways, cardiovascular responses, and cognitive processing in migraine patients. Furthermore, our study's use of brain activity measurements during cognitive tests revealed consistently decreased activity in migraine patients compared with healthy controls. This observation adds to the growing body of evidence suggesting that migraine affects not only pain perception, but also broader aspects of brain function. By integrating these perspectives, we gain a more nuanced understanding of the multisystemic nature of migraine. This emphasizes the need for holistic research methodologies that capture the complex interplay among neurological, autonomic, and cognitive domains. Such an approach is crucial to develop more effective treatments and management strategies for migraine patients, addressing not only pain relief, but also the broader implications for brain functioning and patient quality of life.

Therefore, our results provide valuable insights into the cognitive and autonomic effects of untreated migraine in this specific context, but further research with treated patients is essential for more extensive generalizability. Based on our results and those of others, unmedicated/untreated migraine patients should be carefully treated to avoid future issues. Nevertheless, further studies must be performed to confirm our suggestions and compare early migraine treatments against late medication treatments.

### Study limitations

The current study has some limitations, such as the small sample size, which could have hindered the statistical analysis. In this study, we did not perform sample size calculation or power analysis due to challenges in finding untreated migraine subjects. Given the as the present was a case-control pilot study, such statistical calculations could have rendered the study unfeasible. While we acknowledge this limitation, our research has raised important unanswered questions that warrant further investigation in larger studies. Another limitation was that the participants were recruited with a focus on untreated migraine patients who had received a medical diagnosis of migraine. The decision to include a “young” population was driven by our primary objective of examining cognitive function and autonomic regulation in individuals with migraine who were not undergoing any treatment. During the recruitment process, we observed that a significant proportion of untreated migraine patients fell within the younger age group. This study's focus on a “young” population of untreated migraine patients, coupled with the case-control design wherein healthy individuals were meticulously matched based on age, contributes as a strength. By excluding potential confounders such as age-related comorbidities, we aimed to isolate and investigate the true effects of migraine on cognitive function and autonomic regulation. This approach enhances the internal validity of our findings, enabling a more accurate assessment of the specific impacts of migraine on the aspects studied. The thied limitation was that no subgroup analyses were performed regarding time of migraine or presence of preventive drug treatment. However, it should be noted that this was not the purpose of the present study. Another limitation was that, although we ensured participants were headache-free for at least 5 days before testing, we did not monitor them for migraine occurrence in the 48 hours following the assessments. This means we cannot completely rule out the possibility that some individuals were in the prodromal phase at the time of testing. Future studies should consider postassessment monitoring to better control for this potential confounder. Yet another limitation was that, while the MindWave Mobile device used in this study has been validated to record brain wave variations, its efficacy in clinical conditions, particularly migraine, has not been extensively studied. 24 42 The instrument provides an accessible method to assess brain activity, but the results should be interpreted with caution. Future studies using more sophisticated neuroimaging techniques may be necessary to confirm and expand upon our findings. Despite this limitation, our results provide valuable preliminary insights into brain activity differences between migraine patients and healthy controls, which can guide future research using more advanced methodologies. And the last limitation was the absence of data on mood symptoms and sleep quality, which are important factors that can influence cognitive performance and autonomic regulation in migraine patients. Anxiety, depression, and insomnia are common comorbidities in individuals with migraine, and they have been shown to independently affect cognitive function and autonomic nervous system activity. To address this limitation, future research should incorporate validated instruments to assess these variables, such as the Hospital Anxiety and Depression Scale (HADS) for mood symptoms and the Pittsburgh Sleep Quality Index (PSQI) for sleep quality.

In conclusion, the current study revealed significant differences in brain activity and autonomic regulation between untreated migraine patients and healthy individuals. Despite the lack of significant differences in cognitive test performance between the groups, migraine patients exhibited reduced brain activity variation during cognitive tests. Additionally, they showed an imbalanced autonomic regulation, characterized by decreased sympathetic activity and increased parasympathetic action. These findings suggest that alterations in brain activity and autonomic function occur in migraine patients even in the absence of measurable cognitive deficits.
